# Impact on Body Composition After Two Years of Elexacaftor–Tezacaftor–Ivacaftor Therapy in Children with Cystic Fibrosis

**DOI:** 10.3390/children12121598

**Published:** 2025-11-24

**Authors:** María Álvarez Merino, Concepción Marina López Cárdenes, Saray Mesonero Cavia, Encarnación Torcuato Rubio, Alejandro Rodríguez-Martínez, Saioa Vicente Santamaría, Clara Viñas Torne, Marina Álvarez Beltrán, Celia Gascón Galindo, María Garriga García, Ana Muñoz Alonso, Mercedes Murray Hurtado, Sara Sierra San Nicolás, Pilar Ortiz-Pérez, José Ramón Gutiérrez Martínez, Marta Suárez González, David González Jiménez, Juan José Díaz Martín

**Affiliations:** 1 Pediatrics Department, Central University Hospital of Asturias, 330011 Oviedo, Spain; maria.alvarezme@sespa.es (M.Á.M.); ramongut68@gmail.com (J.R.G.M.); marta.suarezglez@gmail.com (M.S.G.); 2 Cystic Fibrosis Unit, Pediatrics Department, Ramón y Cajal University Hospital, 28034 Madrid, Spain; conchi.95.lc@gmail.com (C.M.L.C.); celiagascon@hotmail.com (C.G.G.); maria.garriga@salud.madrid.org (M.G.G.); 3 Cystic Fibrosis Unit, Vall d’Hebron University Hospital, 08035 Barcelona, Spain; saraymes@gmail.com (S.M.C.); clara.vinas@vallhebron.cat (C.V.T.); marina.alvarez@vallhebron.cat (M.Á.B.); 4 Cystic Fibrosis Unit, Pediatric Gastroenterology and Nutrition Section, Málaga Regional University Hospital, 29010 Málaga, Spain; encatr@gmail.com (E.T.R.); portizp@gmail.com (P.O.-P.); 5 Cystic Fibrosis Unit, Virgen del Rocío University Hospital, 41005 Sevilla, Spain; armgastropediatria@gmail.com (A.R.-M.); anamual@gmail.com (A.M.A.); 6 Cystic Fibrosis Unit, Pediatrics Department, 12 de Octubre University Hospital, 28041 Madrid, Spain; saio.vicente@gmail.com; 7 Pediatric Gastroenterology and Nutrition Section, Pediatrics Department, Canarias University Hospital, 38320 Santa Cruz de Tenerife, Spain; mercedes.murray.hurtado@gmail.com (M.M.H.); sarasierrasannicolas@gmail.com (S.S.S.N.); 8 Pediatric Gastroenterology, Hepatology and Nutrition Unit, Central University Hospital of Asturias, 330011 Oviedo, Spain; juanjo.diazmartin@gmail.com

**Keywords:** cystic fibrosis, CFTR modulators, body composition, bioelectrical impedance, anthropometric parameters

## Abstract

**Background:** Triple therapy with cystic fibrosis transmembrane conductance regulator (CFTR) modulators in patients with cystic fibrosis (CF) has led to a shift in the nutritional management of the disease. Correct assessment of nutritional status is crucial due to its significant impact in pulmonary function and overall patient survival. This study aims to provide mid-term data on anthropometric and body composition changes in pediatric patients treated with elexacaftor–tezacaftor–ivacaftor (ETI). **Methods**: A prospective, longitudinal, multicenter study was conducted involving pediatric CF patients receiving ETI therapy. Anthropometric measurements and bioelectrical impedance analysis (BIA) data were collected at baseline and after 24 months of treatment. **Results**: A group of 66 pediatric patients, median age of 11.11 years (IQR: 8.2–12.5), was studied. The BMI z-score increased from −0.63 at baseline to −0.38 (*p* < 0.001) after 2 years of ETI treatment. Somatometric parameters were compared with a retrospective cohort showing no significant results. Significant changes comparing body composition were also observed over the 24 months period: fat mass (FM) increased from 6.2 kg to 7.4 kg (*p* < 0.001) free fat mass (FFM), increased from 28.2 kg to 34.2 kg (*p* < 0.001) and body cellular mass (BCM), increased from 7.8 kg to 8.7 kg (*p* = 0.02). **Conclusions**: Pediatric patients undergoing ETI treatment showed mid-term improvements in anthropometric parameters, alongside notable changes in body composition. Long-term studies are needed to confirm these findings and to better understand the implications for pediatric CF care.

## 1. Introduction

Cystic fibrosis (CF) is an autosomal recessive genetic disorder caused by mutations in the cystic fibrosis transmembrane conductance regulator (CFTR) gene, identified in 1989 [1,2]. This mutation disrupts chloride transport channels in epithelial cells, leading to thickened secretions that affect multiple organs, including the lungs, pancreas, liver, gastrointestinal tract, and reproductive system [1,3,4]. Traditionally, diagnosis was made in symptomatic children presenting with signs such as recurrent pulmonary infections using sweat chloride testing and genetic analysis [1]. Currently, most cases are identified early and in asymptomatic patients through newborn screening programs [5]. Historically, treatment focused on symptomatic management, including airway clearance therapy, antibiotics, and nutritional support [6,7]. In recent decades, CFTR modulators have emerged, targeting the underlying protein defect [8]. These include potentiators, correctors, amplifiers, and stabilizers, though only the first two classes have been approved for clinical use to date [8]. The triple combination therapy of elexacaftor–tezacaftor–ivacaftor (ETI), approved in 2019, represents a breakthrough due to the significant improvement achieved in pulmonary function and overall survival [9,10,11]. Together with early diagnosis, these advances have transformed the disease course of CF, shifting it from a fatal pediatric condition to a chronic disease with increasing prevalence and improved life expectancy [12,13]. Therefore, these therapeutic advances prompt a reassessment of comorbidities historically associated with CF [10,11]. In the pre-triple therapy era, patients often exhibited chronic malnutrition, mainly due to exocrine pancreatic insufficiency and increased energy demand [1,4]. Both factors are now being effectively addressed by CFTR modulators, supporting the expectation that nutritional status will improve alongside clinical outcomes [14].

Body mass index (BMI) has been used to assess the nutritional status of patients with CF [15,16]. The relationship between BMI and pulmonary function is well-established and commonly used in clinical practice [17,18]. However, with the shift in the nutritional paradigm for these patients, in recent years it has been questioned whether BMI alone accurately reflects true nutritional status, as it does not distinguish between fat mass (FM) and fat-free mass (FFM) [16,19]. Thus, a patient may have a BMI within the recommended nutritional target for the disease, but at the expense of an increased FM and decreased FFM, a situation that is not beneficial for either pulmonary function or the development of other comorbidities, such as cardiovascular events, which are starting to appear in these patients due to increased life expectancy [20,21]. Methods such as bioelectrical impedance analysis (BIA) have been evaluated as tools for measuring nutritional status, as they allow for a reliable determination of body composition (specifically FFM), which is a better marker of malnutrition and, therefore, more closely correlated with pulmonary function in patients with CF [22,23,24,25].

Our working hypothesis is that the nutritional status of patients with CF, assed through both anthropometic measurements and body composition, improves after two years of triple therapy with ETI. Therefore, the aim of this study is to evaluate changes in anthropometric measurements and body composition parameters among pediatric patients with CF following 24 months of treatment with ETI.

## 2. Materials and Methods

### 2.1. Study Design

A longitudinal, prospective, multicenter study was designed involving six tertiary-level hospitals across Spain. The study was approved by the Clinical Research Ethics Committee of Hospital Universitario Ramón y Cajal (Madrid) and subsequently endorsed by the ethics committees of the participating hospitals. Participants and/or their parents or legal guardians were informed about the study objectives and procedures and voluntarily signed the informed consent form specifically prepared for this purpose. A retrospective cohort was constituted, including patients from the same centers who were matched for sex, age range (5–18 years), and prevalence of exocrine pancreatic insufficiency. This cohort was used for comparison with somatometric outcomes after two years of ETI therapy.

### 2.2. Inclusion and Exclusion Criteria

Patients aged between 5 and 18 years diagnosed with CF and eligible for triple therapy with ETI were included in the study. Only clinically stable patients were eligible for inclusion. Patients were excluded if they were lost to follow-up or the BIA was not performed. Clinical instability was defined as the presence of any of the following: platelet count < 50,000/mm^3^, transaminase levels exceeding three times the upper limit of normal, hepatic insufficiency, severe cholestasis (serum bilirubin exceeding two times de upper limit), renal failure (glomerular filtration rate lower than 60 mL/min/1.73 m^2^), recent changes in usual treatment, or hospital admission within the two weeks prior to enrollment.

The results of somatometry after two years of treatment with ETI were compared with a retrospective cohort. Data were obtained from a cross-sectional study published by the same research group, between 2012 and 2014, by González Jiménez D. et al. [26]. Hospitals that did not participate in the current study were excluded and only patients aged between 5 and 18 years were included, resulting in a cohort with the same age range and no differences in gender or exocrine pancreatic function.

### 2.3. Data Collection

Data were collected at treatment initiation and after 24 months. The following groups of variables were included:

1. Demographic data: gender, pubertal status, date of birth, age at each visit.
2. Clinical history of the disease: CF genotype, age at diagnosis, diagnosis through newborn screening, meconial ileus as antecedent, baseline pulmonary function, presence and manifestation of exocrine and/or endocrine pancreatic insufficiency, and associated liver disease, existence, and characterization.
3. Anthropometric data: weight (kg), height (cm), and body mass index (BMI). Anthropometric parameters were calculated using the Nutritional Application of the Spanish Society of Pediatric Gastroenterology, Hepatology, and Nutrition (SEGHNP) https://www.seghnp.org/nutricional/, accessed on 25 March 2025. Spanish growth charts were used as the reference for children older than 6 years [27]. 2006 World Health Organization (WHO) growth standards were applied for those aged 6 years or younger [28]. Results were expressed as z-scores. Malnutrition was defined as BMI below the 10th percentile, nutritional target as BMI between the 10th and the 85th percentile, and overweight as a BMI above the 85th percentile.
4. BIA: FM, FFM, body cellular mass (BCM), total body water (TBW), phase angle (PA). Body composition analysis was performed by monofrequency bioelectrical impedance (50 kHz), with an 8 h fasting period. Participants were assessed while lying in a supine position on a flat, non-conductive stretcher. Arms were positioned approximately 30° away from the trunk, and legs were separated to maintain a minimum distance of 20–45 cm between the ankles, ensuring no contact between the thighs or with conductive surfaces. Electrode placement followed the distal hand–foot tetrapolar method. Four biatrode electrodes were placed on the right side of the body—two on the hand and two on the foot—with the signal electrodes located on the wrist and ankle, and the detector electrodes positioned approximately 5 cm away on the metacarpophalangeal and metatarsophalangeal lines. These electrodes were connected by wire to the bioimpedance device, which measured resistance and reactance. Each participating center used its own BIA model.
5. Pulmonary function: forced expiratory volume in 1 s (FEV_1_) was collected from spirometry assessments. The GLI Calculator (https://gli-calculator.ersnet.org/index.html, accessed on 10 March 2025) was used to interpret the measured lung function values based on the Global Lung Function Initiative (GLI) reference equations [29]. Age, sex, height, ethnicity, and measured FEV_1_ values were added to obtain predicted percentages.

### 2.4. Statistical Analysis

The mentioned data were stored and managed in anonymized form using the REDCap (Research Electronic Data Capture) platform of the SEGHNP [30]. Technical support was provided by the AEGREDCap support unit shared by the Asociación Española de Gastroenterología (AEG). Statistical analysis was performed using STATA software, version 18. Demographic data, CF-related characteristics, anthropometric measurements, and BIA results were expressed as medians, along with their corresponding interquartile ranges. A Shapiro–Wilk test indicated that the sample data did not fit to a normal distribution. Therefore, differences in anthropometric and body composition data were analyzed using the Wilcoxon test, considering a *p*-value < 0.05 as statistically significant. The McNemar test was used to compare pre- and post-treatment anthropometric data.

## 3. Results

### 3.1. Patient Characteristics

The study involved 66 patients (51.5% males). Table 1 shows the frequency distribution of demographic data and the CF-related characteristics of the study population.

### 3.2. Anthropometric Measurements

Data on the evolution of the nutritional status of the patients at baseline and after 24 months of treatment are shown in Table 2.

Nutritional status was assessed by comparing the proportions of patients classified as undernourished, within the nutritional target, or overweight at baseline and after 24 months of triple therapy. However, no statistically significant changes were found. At baseline, 24.2% of patients were within the nutritional target range, compared to 31.8% at 24 months (*p* = 0.26). The percentage of undernourished patients changed from 19.7% to 15.1% (*p* = 0.45), while the proportion of overweight patients was initially 4.5% compared to 7.6% 24 months after treatment (*p* = 0.41). There were no significant differences in somatometric parameters compared with the control group.

Pulmonary function was analyzed at baseline and after 24 months of treatment in a total of 20 patients, showing a statistically significant increase in FEV_1_ values, both in percentage and liters, from 87.9% (65.9–111.9) to 103.1% (86.4–114.8) (*p* = 0.02). When examining the correlation between FEV_1_ improvement and anthropometric parameters, a significant correlation was found only between weight gain (kg) and FEV_1_ (L), r = 0.598 (*p* = 0.005). There was a borderline correlation between the increase in BMI (kg/m^2^) and FEV_1_ (L), r = 0.396 (*p* = 0.08).

### 3.3. BIA Results

Body composition was evaluated at baseline and after 24 months of treatment. Data for BIA results are shown in Table 3 before the start of treatment and again 24 months later using BIA.

A subgroup analysis was carried out, which included gender, CF-related liver disease, CF-related glucose disorder, and pubertal status. Regarding gender-specific analysis, an increase in PA was observed in males, from 6 degrees (5.5–6.8) to 6.1 degrees (5.7–7.3) (*p* = 0.06), and, in females, a decrease in BCM (%) was observed from 50.4 (59–50.4) to 47.8 (54.2–47.7) (*p* = 0.007). In terms of body composition according to CF-related liver disease, patients without liver disease showed a statistically significant decrease in TBW from 62.7% (57–68.6) to 59.6% (57.1–63.7) (*p* = 0.03). Regarding body composition in relation to CF-related glucose disorders, patients with glucose disorder showed a significant increase in FM (kg), from 7.5 kg (4.7–10.1) to 9.3 kg (12.6–6.2) (*p* < 0.001). Additionally, a decrease in TBW was observed in these patients, from 61.1% (58.4–63.5) to 58.9% (55.2–61.6) (*p* = 0.008). Body composition was also analyzed according to pubertal stage, dividing patients (*n* = 49) into prepubertal and pubertal onset groups. Patients undergoing puberty showed a statistically significant increase in FM (kg), from 8.4 kg (6.3–12.4) to 11.9 kg (8.7–11.9) (*p* = 0.02), as well as a significant decrease in TBW (%), from 62.8% (58.4–68.6) to 58.7% (55.2–61.2) (*p* < 0.001).

## 4. Discussion

The introduction of triple therapy with CFTR modulators has resulted in a significant change in the management of the disease for patients with CF [12,13]. Assessing nutritional status is crucial due to its significant impact on pulmonary function and overall patient survival [21,31,32]. Consequently, the optimal method for evaluating the nutritional status of patients with CF receiving triple therapy with CFTR modulators remains a key issue [23]. The present study shows significant mid-term changes in both anthropometric measurements and body composition parameters in pediatric patients treated with ETI for 24 months.

Firstly, focusing on nutritional assessment through anthropometric values, the association between ETI treatment and increased weight, as well as BMI, appears to be clear and well-documented in both adult and pediatric patients [14,20,25,33]. Stewart KL. et al. published, in 2024, the results in a cohort of 154 adult patients after 6 months of ETI treatment [34]. Sutharsan S. et al. included a larger sample of 2645 patients older than 12 years of age, with a follow-up of 12 months [35]. Both studies reported a significant increase in BMI values [34,35]. The results of our study also show a significant increase in both BMI and weight, but after 24 months of treatment, representing a longer follow-up period. Furthermore, our data were obtained in pediatric patients, an age group that is less described in the literature. Additionally, pulmonary function and its potential relationship with nutritional improvement following ETI treatment were evaluated in small sample size. The results suggest a possible association, as previously reported in adult populations [35]. However, as noted, these findings should be interpreted with caution due to the limited sample size.

A retrospective cohort was included, showing no significant differences in BMI. The main explanation is that pediatric patients with CF receive early, intensive, multidisciplinary nutritional support [16]. As a result, many children are able to maintain BMI values close to the recommended targets even before ETI initiation, which reduces the margin for further detectable BMI improvement once ETI is started. This limitation could potentially be addressed by using a prospective cohort or extending the follow-up period.

Regarding body composition, in our pediatric cohort, it would be expected that the results would resemble those observed in the adult population, namely a gain in body mass, predominantly at the expense of FM [25,36]. However, more recent data in pediatric populations suggest a different profile. López-Cárdenes C.M et al. assessed 234 pediatric patients undergoing treatment with ETI using BIA, and a significant increase in both FM and FFM was observed at six months of treatment, indicating a more balanced improvement in body composition [37]. Our results, derived from a prolonged 24-month follow-up, are consistent with these findings. We documented a significant increase in both FM and FFM, as well as an increase in BCM. Furthermore, although the improvement in PA did not reach statistical significance, a positive trend was noted, which may reflect improvements in cellular membrane integrity and overall nutritional status. These findings were further explored through subgroup analyses based on gender, CF-related liver disease, CF-related glucose disorder, and pubertal status. Observations such as a significant increase in fat mass (FM) in patients with glucose disorders, as well as in those undergoing puberty, suggest heterogeneity in treatment response and its potential influence on the magnitude and pattern of body composition changes in pediatric patients receiving ETI.

Studies evaluating BIA in patients with CF primarily focus on FM and FFM due to the proven impact of FFM in pulmonary function, but our findings suggest that a broader BIA profile may provide added clinical value [17,19]. BIA provides insights beyond FM and FFM; parameters such as PA and BCM serve as indicators of metabolically active tissue and cellular health [38]. PA has demonstrated prognostic value across multiple pathologies, as described in the review by Norman K et al. [39]. Also, it has been identified as an independent predictor of mortality in critically ill patients [40]. The more balanced body composition profile observed in our pediatric cohort may reflect several contributing factors. These could include the fact that children, being in a phase of active growth, may adapt more effectively than adults. The finding of an increase in FM (kg), predominantly among pubertal patients, is consistent with this statement and with previously reported findings in adult patients [41]. Additionally, children are typically managed from an early age in specialized units with expertise in nutrition and dietetics. These patients, therefore, have been educated from early on about the current dietary approach to CF, which emphasizes a balanced and high-quality diet rather than prioritizing caloric intake above all else [42]. The 2024 consensus guidelines by Wilschanski M et al. reinforce this approach, recommending dietary diversity and moderation in fat and protein intake, along with standardized methods for monitoring body composition [16].

A key strength of this study is the 24-month follow-up period, the longest reported in pediatric cohorts receiving ETI therapy. The medium-term follow-up allows us to state that the increases in BMI, FM, and weight observed at earlier stages by López-Cárdenes C.M et al. were maintained over time, without evidence of disproportionate or accelerated gains [37]. Another strength lies in the sample size: to our knowledge, this is the largest pediatric cohort assessed for body composition changes over a prolonged treatment period.

On the other hand, we must point out some limitations of our study. Some data loss occurred during follow-up due to patient dropout, particularly among those who had only recently initiated ETI. As an observational study design without a control group, causality cannot be established. To partially address this significant limitation, we included a historical cross-sectional cohort to strengthen the study design, while acknowledging that an ideal historical cohort would have been prospective or longitudinal. Although this was a mid-term follow-up study, a longer observation period using a larger sample size may be necessary to detect more substantial changes as in nutritional status classification. Moreover, as the participants were children undergoing normal growth, these changes cannot be attributed only to ETI. Additionally, there are several limitations regarding the BIA results. The specific BIA models used were not standardized across participating centers, and the substantially smaller sample size for several BIA outcomes may have limited the statistical power to detect significant differences or associations. An additional limitation of the study is the lack of complete data on acute exacerbations and hospitalizations, as well as other variables such as physical activity and additional lifestyle factors. Future studies should address this aspect given its clinical significance and potential to reinforce the evidence on the overall benefits of triple modulator therapy. Despite these limitations, our findings offer valuable contributions to the evolving understanding of nutritional responses to CFTR modulators in pediatric patients. They highlight the relevance of body composition analysis—beyond traditional anthropometric measures—as an essential tool for optimizing individualized care in the era of highly effective CF therapies.

## 5. Conclusions

CFTR modulator therapies have brought about a paradigm shift in the clinical management of CF [8,41]. From a nutritional perspective, the traditional concern regarding malnutrition is increasingly being replaced by issues related to overweight and obesity, particularly in adult patients [14,17,25]. In this context, tools such as BIA offer valuable insights into changes in body composition and complement conventional anthropometric assessments, allowing for a more individualized and dynamic approach to patient management [16,23]. In our study, pediatric patients undergoing treatment with triple therapy using CFTR modulators demonstrated mid-term improvements in anthropometric parameters, alongside notable changes in body composition.

## Figures and Tables

**Table 1:** Demographic and FQ-related characteristics.

Category	Results
Gender (*n* = 66)	
Male, *n* (%)	34 (51.5)
Female, *n* (%)	32 (48.5)
Pubertal status (*n* = 49)	
Prepubertal, *n* (%)	28 (57.1)
Tanner stage 2, *n* (%)	1 (2)
Tanner stage 3, *n* (%)	2 (4.1)
Tanner stage 4, *n* (%)	3 (6.1)
Tanner stage 5, *n* (%)	15 (30.6)
Genotype (*n* = 66)	
Homozygous DF, *n* (%)	24 (36.4)
Heterozygous DF, *n* (%)	40 (60.6)
Mutation other than DF	2 (3)
Study onset age (y) Md (IQR)	11.11 (8.2–12.5)
Neonatal screening diagnosis (*n* = 66)	
Yes, *n* (%)	41 (62.1)
No, *n* (%)	25 (37.9)
Meconial ileus (*n* = 66)	
Yes, *n* (%)	5 (7.6)
No, *n* (%)	61 (92.4)
Diagnosis age (m) Md (IQR)	18 (5–48)
Pancreatic exocrine insufficiency (*n* = 66)	
Yes, *n* (%)	58 (87.9)
No, *n* (%)	8 (12.1)
Units of lipase (U/kg/day) Md (IQR)	6100 (4300–7537)
Baseline pulmonary function (*n* = 56)	
FEV_1_ (%)	82.7 (70.4–99.2)
FEV_1_ (L)	1.9 (1.3–2.2)
CF-related liver disease (*n* = 66)	
No, *n* (%)	47 (71.2)
Yes, *n* (%)	19 (28.8)
Liver disease without cirrhosis, *n* (%)	17 (25.8)
Multinodular cirrhosis, *n* (%)	2 (3)
CF-related glucose disorder (*n* = 66)	
No, *n* (%)	35 (53)
Yes, *n* (%)	31 (47)
Impaired fasting glucose (IFG), *n* (%)	1 (1.5)
Glucose intolerance, *n* (%)	12 (18.2)
CFRD without IFG, *n* (%)	2 (3)
CFRD with IFG, *n* (%)	7 (10.6)
Indeterminate glucose tolerance, *n* (%)	9 (13.6)

Results are expressed as absolute numbers and percentages or as median and its interquartile ranges (Q1–Q3), as appropriate. Abbreviations: *n*, total; DF, F508del; y, years; Md, median; IQR, interquartile range; m, months; FEV_1_, Forced Expiratory Volume in 1 s; L, liters; UI/kg/d, units per kilogram per day; IFG, impaired fasting glucose; CFRD, cystic fibrosis-related diabetes.

**Table 2:** Comparison of anthropometric measurements results before and after 24 months of ETI.

Anthropometric Values	0 Months (*n* = 66)	24 Months (*n* = 66)	*p*-Value *	Control Group (*n* = 67)	*p*-Value **
Weight					
kg	32.5 (23.8–43.2)	40.9 (52.2–30.6)	<0.001	35.4 (28.3–50.9)	0.321
z-score	−0.79 (−1.2, −0.3)	−0.54 (−1.1, −0.01)	<0.001		
Height					
cm	142.5 (128–156)	150.2 (134.1–159)	<0.001	145 (128.5–162)	0.298
z-score	−0.53 (−1.1, −1.1)	−0.62 (−1.1, −0.16)	0.36		
BMI					
kg/cm^2^	16.1 (15.1–18.3)	17.9 (16.1–20.4)	<0.001	17.8 (16.0–19.6)	0.564
z-score	−0.63 (−1.1, −0.04)	−0.38 (−0.88, −0.08)	<0.001	−0.37 (−0.67, −0.12)	0.407

Results are expressed as median and its interquartile ranges (Q1–Q3). * *p*-values were estimated using the Wilcoxon signed-rank test. ** *p*-values were estimated using two-sample Wilcoxon rank-sum test, and the 24 months and control groups.

**Table 3:** Comparison of BIA results before and after 24 months of ETI.

BIA Parameters	0 Months	24 Months	*p*-Value *
FM (kg) (*n* = 66)	6.2 (3.9–9.2)	7.4 (5–11.9)	<0.001
FM (%) (*n* = 66)	19.5 (15.1–23.3)	19.3 (16.1–24.1)	0.45
FMI (*n* = 29)	2.9 (2.2–4.3)	3.4 (2.5–4.6)	0.41
FFM (kg) (*n* = 66)	28.2 (19.3–34.1)	34.2 (24.6–40.2)	<0.001
FFM (%) (*n* = 66)	80 (76.4–84.6)	80.7 (75.9–83.7)	0.49
FFMI (*n* = 30)	13.5 (12.4–15.4)	15 (13.5–16.5)	<0.001
BCM (kg) (*n* = 26)	7.8 (7–10)	8.7 (7.3–10.4)	0.02
BCM (%) (*n* = 41)	47.1 (23.4–53.2)	44.8 (26.8–53.5)	0.22
PA (deg) (*n* = 63)	6 (5.4–6.9)	6.1 (5.6–7.2)	0.25
TBW (%) (*n* = 61)	62.6 (58.6–67.2)	61.2 (57.1–66.5)	0.06

Results are expressed as median and its interquartile ranges (Q1–Q3). * *p*-values were estimated using the Wilcoxon signed-rank test. Abbreviations: FM, fat mass; FMI, fat mass index; FFM, free fat mass; FFMI, free fat mass index; BCM, body cellular mass; PA, phase angle; deg, degrees; TBW total body water.

## Data Availability

The original contributions presented in the study are included in the article; further inquiries can be directed to the corresponding author.

## Abbreviations

The following abbreviations are used in this manuscript:

CFTR	Cystic fibrosis transmembrane conductance regulator
CF	Cystic fibrosis
ETI	Elexacaftor–tezacaftor–ivacaftor
BMI	Body mass index
FM	Fat mass
FFM	Fat-free mass
BIA	Bioelectrical impedance analysis
SEGHNP	Spanish Society of Pediatric Gastroenterology, Hepatology, and Nutrition
WHO	World Health Organization
BCM	Body cellular mass
TBW	Total body water
PA	Phase angle
FEV_1_	Forced Expiratory Volume in 1 s
GLI	Global Lung Function Initiative
RED-Cap	Research Electronic Data Capture
IQR	Interquartile range
IFG	Impaired fasting glucose
CFRD	Cystic fibrosis-related diabetes
FMI	Fat mass index
FFMI	Fat-free mass index
